# Solid Lipid Particles for Lung Metastasis Treatment

**DOI:** 10.3390/pharmaceutics13010093

**Published:** 2021-01-13

**Authors:** Lourdes Valdivia, Lorena García-Hevia, Manuel Bañobre-López, Juan Gallo, Rafael Valiente, Mónica López Fanarraga

**Affiliations:** 1 Nanomedicine Group, University of Cantabria—IDIVAL, Herrera Oria s/n, 39011 Santander, Spain; lourdes.valdivia@unican.es (L.V.); lgarcia@idival.org (L.G.-H.); rafael.valiente@unican.es (R.V.); 2 Advanced (Magnetic) Theranostic Nanostructures Laboratory, Nanomedicine Unit, International Iberian Nanotechnology Laboratory (INL), Av. Mestre José Veiga s/n, 4715-330 Braga, Portugal; manuel.banobre@inl.int (M.B.-L.); juan.gallo@inl.int (J.G.); 3 Applied Physics Dept, Faculty of Sciences, Avda. de Los Castros 48, 39005 Santander, Spain

**Keywords:** nanomedicine, cancer, doxorubicin, melanoma, drug delivery

## Abstract

Solid lipid particles (SLPs) can sustainably encapsulate and release therapeutic agents over long periods, modifying their biodistribution, toxicity, and side effects. To date, no studies have been reported using SLPs loaded with doxorubicin chemotherapy for the treatment of metastatic cancer. This study characterizes the effect of doxorubicin-loaded carnauba wax particles in the treatment of lung metastatic malignant melanoma in vivo. Compared with the free drug, intravenously administrated doxorubicin-loaded SLPs significantly reduce the number of pulmonary metastatic foci in mice. In vitro kinetic studies show two distinctive drug release profiles. A first chemotherapy burst-release wave occurs during the first 5 h, which accounts for approximately 30% of the entrapped drug rapidly providing therapeutic concentrations. The second wave occurs after the arrival of the particles to the final destination in the lung. This release is sustained for long periods (>40 days), providing constant levels of chemotherapy in situ that trigger the inhibition of metastatic growth. Our findings suggest that the use of chemotherapy with loaded SLPs could substantially improve the effectiveness of the drug locally, reducing side effects while improving overall survival.

## 1. Introduction

In cancer, surgery is the treatment of choice. However, it is often not an option because many cancer cells have already escaped from the primary tumor and colonized distant tissues. Most patients with advanced metastatic disease confront a terminal illness. In fact, metastasis is the greatest challenge to a cancer patient’s survival. There are currently no effective treatments to stop or prevent this process, so there is an urgent need to find new diagnostic and therapeutic approaches. To inhibit metastasis, local treatment is generally complemented by radiation therapy and high doses of chemotherapy that cause numerous side effects [1,2], limiting the success of metastasis treatment [3]. However, systemically applied cytotoxic drugs are not effective in preventing the spread of metastatic cells that cause 90% of cancer deaths [4].

Melanoma is an example of a fatal malignancy with rapid systemic dissemination. The 5-year survival rate for metastatic melanoma is less than 15%, and the median survival after developing pulmonary metastasis is on average 7.3 months [5]. The high recurrence of the tumor (one-third of all patients) and the low survival rate are due to the failure of chemotherapy as a systemic treatment for metastasis [6]. Recently, hormone therapy and immunotherapy have produced effective results boosting cell-mediated innate and adaptive antitumor immunity [7,8]. Thus, while understanding the molecular mechanisms behind cancer cell invasion of distal vital organs [1,9], new treatments must be developed to prevent/reduce the rate of metastasis.

One possible solution to this issue could be the use of encapsulated drugs in nanosystems to reduce the systemic toxicity and improve the local effect and drug stability through a sustained and controlled drug release. In this sense, nanotechnology can greatly contribute by the development of delivery nanosystems which can inhibit primary tumors and, at the same time, prevent metastases before they sprout. Nanomedicine offers interesting opportunities to design different drug delivery systems, such as liposomes [3], carbon nanotubes [10], or gold nanoparticles [11], among others. These nanosystems can help in (i) reducing the toxicity of the treatment, (ii) preventing the premature elimination or degradation of the therapeutic compound, and (iii) significantly modifying the biodistribution of the encapsulated drug [12,13,14,15]. In this way, drugs with extraordinary pharmacological interest, but discarded due to their poor pharmacological properties or high systemic toxicity, can be reformulated into new nanocomposites showing multifunctional properties and improved therapeutic outputs [16]. Here, we have encapsulated doxorubicin (DOX) in lipidic particles. This drug has already been encapsulated in liposomes in previous studies that have been approved by the U. S. Food and Drug Administration (FDA) (Doxil^®^ [16], Myocet^®^ [17], and LipoDox^®^ [18]). The advantages of these formulations are mainly to do with toxicity. They have less severe side effects than the free drug.

Numerous types of nanocarriers that allow the encapsulation of drugs to be systemically administered have been described. Among these, the most employed are liposomes [3,6], dendrimers [12,13,14], polymeric micelles [5,6,7,8], and silica-based materials [19,20,21]. Most of these nanomaterials have similar drug release patterns. In most examples, encapsulated therapeutic compounds are released at once, upon detachment, degradation, or permeabilization of the nanocarrier and/or its seal [22,23]. This “burst-release effect” triggers a fast peak of drug activity at the local or systemic level that closely mimics the effect caused by the free drug. Unfortunately, the effect of the drug is not sustained over time, selecting surviving cells and so generating resistant clones able to travel and colonize distant tissues. Thus, the development of drug carriers providing an initial delivery together with a sustained and controlled therapeutic release for long periods to obtain a level of drug higher than the minimum effective concentration is highly desirable. This suggests that the design of two-stage-release drug delivery systems could improve the prevention of metastasis.

Here, we used solid lipid particles (SLPs), which are aqueous colloidal dispersions with a matrix composed of solid biodegradable lipids. These nanoformulations present several advantages compared with other nanovehicles. Among the advantages, they show great biocompatibility, high drug-loading capacity, improved pharmaceutic stability, and excellent reproducibility [24]. Furthermore, the natural carnauba wax chosen in this study represents one of the best options to obtain excellent drug encapsulation efficiencies while maintaining the plasma drug concentration within the therapeutic window during a prolonged period [25].

In this study, we have investigated the antimetastatic effect of carnauba wax DOX-loaded SLPs. This drug is the first-line treatment for a wide range of cancers including lymphomas, leukemias, and solid tumors in the bladder, breast, stomach, lung, and ovaries, among others [26]. However, DOX use is currently decreasing due to its side effects, which include cardiotoxicity and nephrotoxicity [27,28]. This encapsulation system has already proved its efficacy in vitro in malignant melanoma cell cultures (2D and 3D melanoma models) [29] compared to the free DOX. Herein, we have studied the in vivo efficacy of the encapsulated DOX in a melanoma metastatic model.

## 2. Materials and Methods

### 2.1. Synthesis and Characterization of SLPs

The synthesis of SLPs was made by a modified melt emulsification method (Figure S1) as previously described [29]. Briefly, 100 mg of carnauba wax (T1 E00018; Koster Keunen, Watertown, CT, USA) was mixed with (i) a total of 30 mg of iron oxide (Fe_3_O_4_) nanoparticles, prepared following the coprecipitation method [30], with 13.02 ± 0.24 nm of diameter in a chloroform solution (99.5%, C2432; Sigma, St. Louis, MO, USA); (ii) 250 μL of 3,3′-dioctadecyloxacarbocyanine perchlorate (DiO) (D275; Invitrogen, Waltham, MA, USA) at 1 mg/mL in chloroform; and (iii) DOX (A14403; Adooq Bioscience, Irvine, CA, USA) (20 mg/mL). This mixture was heated until complete wax melting and chloroform (C2432; Sigma, Darmstadt, Germany) evaporation was achieved. Then, 2.25 mL of Milli-Q water and 250 μL of a 50 mg/mL water solution of Tween80 (P4780; Sigma, Darmstadt, Germany) were added to the vial. The sample was ultrasonicated (Branson 250; Emerson, St. Louis, MO, USA) at 25% power for 2 min at 20 s working intervals. The vial was cooled by a digital sonifier by immersion in ice to solidify the lipid particles. Finally, the formulation was centrifuged (Hettich Zentrifugen Universal 320, Tuttlingen, Germany) (3000 rpm, 10 min) and the supernatant was freeze-dried in the presence of sucrose (0.9% *w*/*w*) as a cryoprotectant.

Hydrodynamic diameters, ζ potential values and the polydispersity index of the SLPs were measured using dynamic light scattering (DLS) (Horiba Scientific SZ-100 instrument; Kyoto, Japan), and morphology was observed through transmission electron microscopy (TEM) images using a JEOL JEM-2100 (Tokyo, Japan) microscope at an accelerating voltage of 200 kV (Figure S2). The amount of DOX loaded from the SLPs was determined by high-performance liquid chromatography (UHPLC; Agilent 1290 Infinity II LC System; Santa Clara, CA, USA) using a gradient of water:acetonitrile (from 100% to 25:75%) and an Aeris 1.7 μm peptide XB-C18 column (Phenomenex; Torrance, CA, USA). Graphics were designed by BioRender.com.

### 2.2. Murine Malignant Melanoma Cells

Murine malignant melanoma B16-F10 cells (ATCC CRL-6475) were grown in Iscove’s Modified Dulbecco’s Medium (IMDM; P04-20350; Panbiotech; Aidenbach, Germany) supplemented with 10% fetal bovine serum (FBS; 26140079; Fisher Scientific; Waltham, MA, USA) and antibiotics. Cells were incubated under 37 °C with an atmosphere of 5% CO_2_. B16-F10 cells were treated at a final concentration of 2 μg/mL of DOX-loaded SLPs (SLPs-DOX) for 2, 16, or 48 h. For confocal fluorescence imaging purposes, cells were fixed with 4% paraformaldehyde (43368; Alfa Aesar; Haverhill, MA, USA) and were immunolabeled with the anti-α-tubulin (B512; Sigma; Germany) antibody to recognize the microtubules. A secondary goat anti-mouse 647 (A21236; Invitrogen; USA) was used. Fluorescent images were obtained with a Nikon A1R confocal microscope (Tokyo, Japan). All images were pseudocolored.

### 2.3. In Vitro Drug Release

DOX release profile was studied in triplicate using dialysis (14 kDa cut-off cellulose membranes; Sigma; Germany) in phosphate-buffered saline media (PBS, pH 7.4). A total of 0.3 mL of DOX-loaded SLPs (SLPs-DOX) dispersion at a concentration of 20 mg/mL of DOX were added into the membrane bags that were submerged in PBS and stirred at 300 rpm at ca. 37 °C. The dialysis membrane allowed drug diffusion into the PBS while retaining the SLPs. Samples of 1 mL were taken at different times and measured using fluorescence spectroscopy in an Edinburgh Inst. FLSP920 spectrofluorometer (Livingston, UK). Values were compared to those of the calibration curve prepared for DOX (Figure S3). Finally, DOX release data in PBS pH 7.4 were fitted to a Korsmeyer–Peppas kinetic model.

### 2.4. Animal Studies, In Vivo Model

In vivo experiments were designed and performed to minimize the use of animals. C57BL/6 mice (12–16 weeks old) were housed with a 12 h light/dark cycle with free provision of food and water at the Experimentation Service (SEEA) of the University of Cantabria. Animals were maintained, handled, and sacrificed following the directive 2010/63/UE. The B16-F10 lung metastasis model in the C57BL/6 strain of mice is well established in the literature [31,32,33,34]. This cell line was derived from a spontaneous melanoma developed in C57BL/6 mice [31]. Metastatic foci were produced upon intravenous B16-F10 malignant melanoma cell transplantation. For this purpose, ca. 100,000 B16-F10 melanoma cells (50 μL/mouse) were injected in the retro-orbital venous sinus using a 0.3 mL microsyringe (BD Micro-Fine^TM^; USA). Ten days after intravenous cancer cell injection, mice were randomly divided into four groups: untreated controls, mice injected with free DOX, mice injected with control SLPs, and finally, mice injected with SLPs-DOX. Animals were treated 3 times every 2 days at a concentration of 2.5 mg/kg DOX (each doses). Mice were euthanized 20 days after the transplant and had their tissues collected and fixed in formalin for histology. Lung metastatic colonies were easily recognized as black spots on the lung surface [35]. Histopathological evaluation was performed on hematoxylin (1.09249.0500; Merck; Kenilworth, NJ, USA) and eosin (256879; Panreac; Barcelona, Spain) stained paraffin-embedded lung tissue sections. Graphics were designed by BioRender.com.

### 2.5. Quantification of Metastasis and Statistical Analysis

The metastasis affection in the lungs was quantified using ImageJ software. Upon lung extraction, the lungs were photographed, and lesions were quantified automatically using the ImageJ software. Values were compared using a Student’s two-tailed *t* test statistical analysis. The significance was established for (*) *p* = 0.001. The total number of events and the confidence levels obtained in the experiment (*n*) are all indicated in the figure captions. Results are expressed as mean values with their corresponding standard errors.

### 2.6. In Vivo SLP Distribution

The Fe_3_O_4_ nanoparticles in the nanocarrier allowed the determination of the SLP distribution in the different tissues using inductively coupled plasma optical emission spectrometry (ICP-OES) (ICPE-9000 SHIMADZU; Kyoto, Japan). For this purpose, tissues of 4 mice per treatment were collected at 20 days postinjection, fixed in formalin, weighed, and calcined at 450 °C for 12 h to remove all the organic matter. The obtained ashes were dispersed in concentrated chlorhydric acid (HCl 37%) overnight. Finally, samples were diluted to 10 mL with Milli-Q water and analyzed via ICP-OES.

Confocal microscopy imaging was used to visualize particles in lungs 3 and 8 h after intravenous administration. They were embedded in tissue-Tek^®^ O.C.T (optimum cutting temperature) solution (Sakura, Japan), frozen, and cut into 15 μm sections with cryostat. Sections were then fixed in paraformaldehyde (4%) and stained with Hoechst (33258; Sigma; Germany).

## 3. Results

### 3.1. Preparation and Characterization of the Control and DOX-Loaded SLPs

**Figure 1:**
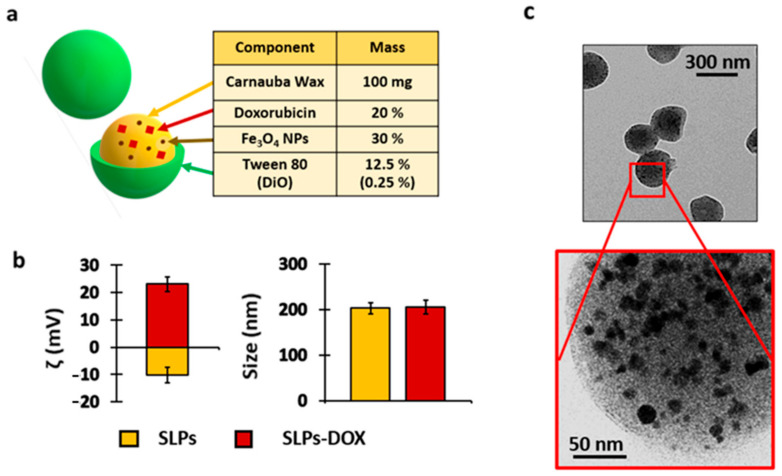
Solid lipid particle (SLP) characterization. (**a**) Composition of the SLPs. (**b**) Hydrodynamic particle size (right) and ζ potential (left) of the control SLPs (yellow) and SLPs loaded with doxorubicin (DOX) (red). (**c**) Representative TEM micrographs of SLPs. The Fe_3_O_4_ nanoparticles are visible as dark structures inside the composite (inset).

The SLPs were synthesized using the melt emulsification method described in the Materials and Methods section (Figure S1). The hydrophobic organic matrix of the SLPs consisted of carnauba wax containing iron oxide nanoparticles. The wax matrix chosen is a natural complex wax obtained from a Brazilian palm tree widely used in food [36], pharmaceutical applications [8], and other technologies [37]. The SLPs were labeled with a fluorescent dye (3,3′-dioctadecyloxacarbocyanine perchlorate (DiO)), which was used for detection and imaging purposes. DiO has been widely used due to its biocompatibility and great stability in living and fixed tissues [38]. This dye allows particle localization using fluorescence technologies in processed cells and tissues [39]. The encapsulation of iron nanoparticles inside the wax matrix allowed us to know the biodistribution of SLPs in in vivo studies. Concentrations of iron were measured in different tissues of mice by the ICP technique. In this study, particles were loaded with 20% (*w*/*w*) of DOX to study if a drug delivery system can decrease the toxicity and improve the antineoplastic and antimetastatic effects of traditional chemotherapy (Figure 1a).

Upon SLPs-DOX synthesis, particle characterization revealed a DOX and Fe_3_O_4_ nanoparticle encapsulation efficiency of 86.5% ± 1.4 and 99%, respectively. The SLPs presented a hydrodynamic diameter of ca. 200 nm and an estimated ζ potential value of −10 and +23 mV for the control SLPs and SLPs-DOX, respectively. This charge change can be attributed to the positive charge of DOX at physiological pH (Figure 1b). The values of the polydispersity index were 0.36 ± 0.04 for the control SLPs and 0.16 ± 0.1 for the SLPs-DOX (Figure S2). Electron microscopy images show the rounded shape of particles. This technique also allowed the identification of the iron oxide nanoparticles inside the wax matrix (Figure 1c, dark hypointense spots).

### 3.2. Lung Targeting of SLPs

It is described that SLPs present several advantages in the treatment of pulmonary pathologies, stemming from their adequate size, the potential for deep lung deposition, low toxicity, and prolonged drug release [40].

To study the biodistribution of SLPs after intravenous injection (Figure S3), we administered in lung tissues fluorescent control SLPs particles and analyzed the presence of DiO fluorescence in lung tissue cryosections using fluorescence confocal microscopy. Figure S3a shows confocal Z-projection images of lung slides from SLP-treated mice. The particles are identified as small green spots in the vicinities of the nuclei of the cells. Particles were identified in the lung tissue at both 3 and 8 h after intravenous injection. To confirm this lung distribution after a long period (20 days after injection), we performed an additional biodistribution test quantifying the iron content by ICP-OES. This analysis revealed a broad distribution in the analyzed organs but also lung accumulation of the particles 20 days after injection (Figure S3b). Together, these results confirm that SLPs targeted the lung tissues.

### 3.3. A Two-Stage Drug Release Effect of SLPs

A few nanocarriers have been proposed capable of transporting and releasing an encapsulated drug upon activation by different stimuli. Two approaches can be adopted in designing stimuli-responsive drug nanosystems. In one approach, endogenous stimuli can be exploited for enhancing drug release. These include pH or enzymatic degradation [41]. This effect requires the selection of appropriate materials for designing the nanocarriers, which should respond to a specific endogenous stimulus releasing all of the encapsulated drug simultaneously (the so-called “burst release”). In the second approach, physical stimuli are applied externally to targeted tissue after administration of drug-loaded specific nanocarriers. These exogenous stimuli include temperature, light, magnetic field, electric field, and ultrasound [42]. The application of these exogenous stimuli is responsible for the alteration of the structure of specifically designed nanocarriers, which leads to drug release at targeted tissues [43,44]. In some nanosystems such as liposomes [45], the drug is released in two steps. The first is upon contact with blood, releasing the drug adsorbed on their surfaces producing a “burst-release effect” that triggers a peak of drug activity at the local or systemic level that is somewhat similar to the effect seen when the free drug is administered. During the second phase, the intraparticle cargo is released.

**Figure 2:**
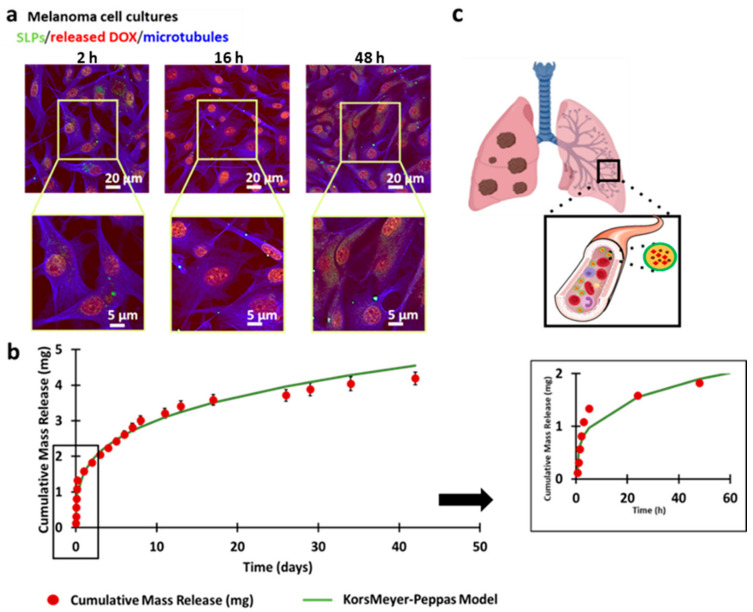
DOX release from DOX-loaded SLPs (SLPs-DOX) in cultured cells and in vitro. (**a**) Fluorescence confocal microscopy projection images of malignant melanoma cells treated with SLPs-DOX for 2, 16, and 48 h. The SLPs appear in the green channel, and microtubules are shown in the blue channel. The red nuclear fluorescence is indicative of the presence of released DOX. (**b**) In vitro drug release profile of the SLPs-DOX particles in physiological conditions (PBS at 37 °C). The results are mean ± SEM of three experimental replicas. (**c**) Scheme of treatment with SLPs-DOX against metastasis in the lungs.

To investigate the effect of these DOX-loaded SLPs in vitro, we used malignant melanoma cells. Fluorescence confocal microscopy imaging of the cultures revealed that the specific DOX fluorescence progressively accumulated in the cell nucleus during the first 48 h (Figure 2a, red channel), showing an efficient release of drug from the SLPs in the cells.

Drug release from SLPs-DOX was also quantified in vitro (Figure S4). To that purpose, 0.3 mL of SLPs-DOX was resuspended in PBS at a concentration of 20 mg DOX/mL and incubated in rotation at 37 °C inside dialysis membranes. Figure 2b shows how DOX-loaded SLPs present an initial burst-release phase, where ca. one-third of the encapsulated drug is released in the media during the first 5 h. After this initial step, a pattern of prolonged and sustained drug release was observed that lasted for more than 40 days. Drug release data were fitted to a Korsmeyer–Peppas model [46]. The release profile of DOX can be explained by this model, with a release exponent value of 0.28 and *R*^2^ value of 0.96. The mechanism of drug release confirmed that the SLP drug release mechanism was diffusion (Fickian model) with a slope of <0.5. These release results are consistent with other systems based on lipid matrix particles described elsewhere [47,48,49].

### 3.4. Inhibition of Metastasis Growth or Antimetastatic Efficacy In Vivo by SLPs-DOX

**Figure 3:**
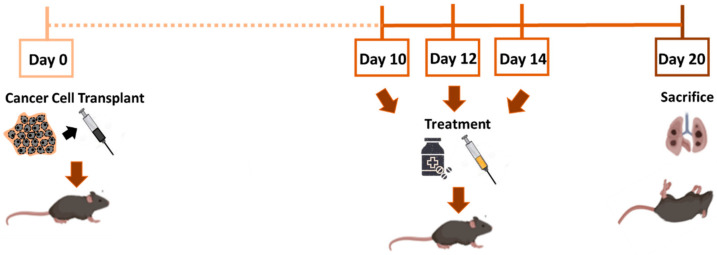
Schematic representation of the metastasis growth inhibition experiment.

**Figure 4:**
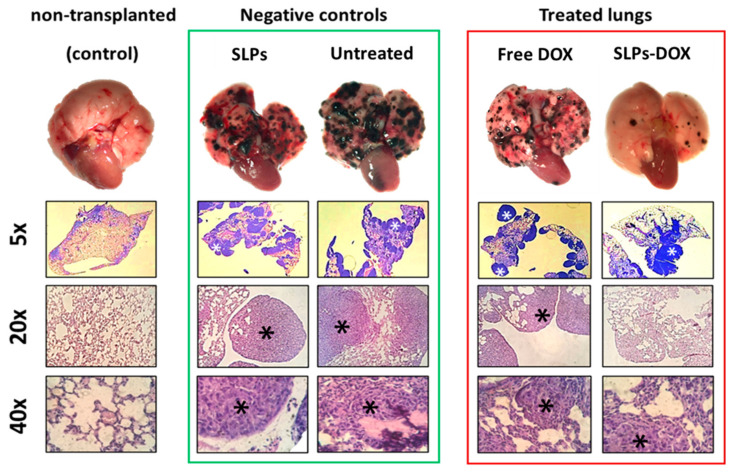
Representative images of metastatic lungs for each experimental group. In the first row, metastatic foci are recognized as black spots on the fresh lung surface. Hematoxylin–eosin-stained histological sections of the lung tissues are shown. Metastatic tissues are indicated by asterisks.

To investigate the ability of SLPs-DOX to inhibit melanoma metastasis in the lungs, we used a well-established mouse metastatic model using B16-F10 melanoma cells as described in Section 2.4. For the study, metastasis-bearing animals were treated intravenously with 3 doses of 2.5 mg/kg of free DOX, or the equivalent amount of encapsulated drug in SLPs-DOX, at 10, 12, and 14 days after metastasis induction (Figure 3). Animals were sacrificed 20 days after the initial cell transplant. Lungs were collected, photographed, and preserved by fixation in formalin. After 20 days of intravenous injection of the cells, black colonies of metastatic cells were clearly observable on the surface of the lungs of all injected mice (Figure 4, negative control).

Figure 4 shows a representative example of the lungs after the different treatments. DOX-treated lungs (whether free or SLPs-DOX) present less metastatic spots than negative controls. In fact, SLPs-DOX-treated lungs present the lowest amount of metastasis. Histological analysis corroborates the results from fresh lung images.

**Figure 5:**
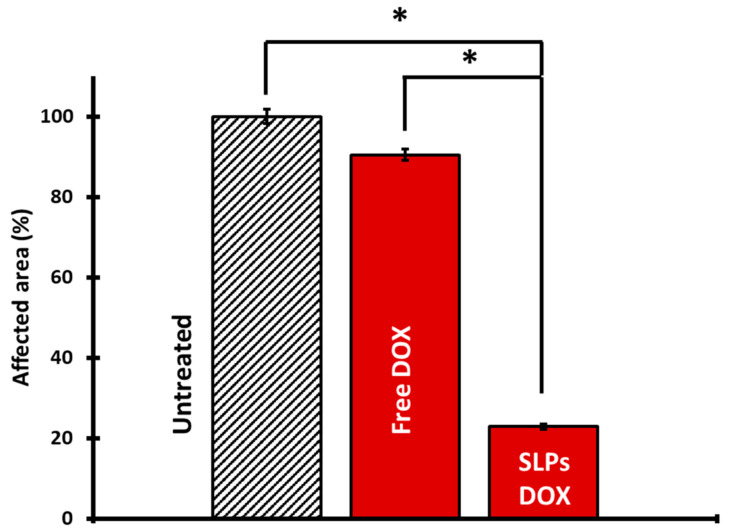
SLPs significantly reduce metastasis in the lungs. Representation of lung metastasis is expressed as the affected area with respect to the total area of the lung. Reduction in metastasis can be observed when the lungs are treated with the SLPs-DOX compared with the lungs treated with the free drug (*t* = 5.94; *n* = 170; * t_0.001_). These differences are also significant between the lungs treated with SLPs-DOX with respect to untreated lungs.

ImageJ software was used to quantify the affected area in the lungs to evaluate the effect of treatment on metastasis. The ratio of affected area/total lung area was calculated in 120 animals. Figure 4 shows representative images of the lungs of mice treated with SLPs-DOX, where approximately a 60% reduction of the affected area was observed compared with mice treated with free DOX. These data, together with the differences between the SLPs-DOX-treated mice and the untreated mice, are statistically significant (Figure 5). It is also important to note that no significant differences were found in the survival rates (Figure S5) or body weight (Figure S6) between mice from the different treatment groups, indicating no detectable SLPs-DOX in vivo toxicity during the timeframe of these tests.

## 4. Discussion

Considerable therapeutic effort in oncology has been focused on stopping cancer growth. Currently, patients with advanced metastasis have a low probability of recovery because there are no treatments to stop or prevent this process. Conventional drug systems and conventional drug carrier production methods have several limitations, such as frequent dosing, poor bioavailability, or poor patient compliance [50]. These formulations are designed in such a way that the therapeutic concentration of the drug must always be within the therapeutic window while trying to avoid the toxic effects that are produced by an overdose [51].

For this purpose, it is essential to study the drug concentration level in blood. On one hand, a high single dose of the drug could cause toxic side effects. On the other hand, lower administrated doses at different times can maintain the drug concentration in plasma, but without an efficient response from the clinical point of view. Therefore, targeted and maintained frequent drug administration with low doses could be a possible solution. In this scenario, smart delivery systems can make a significant contribution. The benefits of such delivery systems are (i) lower dosing frequency dictated by the matrix that releases the drug at a predetermined rate, (ii) higher bioavailability, (iii) improved drug stability, (iv) reduced toxic effect of the drug due to chronic and repetitive use, and (v) reduced drug loss due to continuous elimination [52]. In this context, there are numerous types of drug delivery nanocarriers but there are also many issues associated with them: toxicity, low encapsulation efficacy, lack of drug stability, inability to encapsulate more than one drug, or rapid and ineffective release.

The SLPs chosen in this work are stable, inert, and safe, and most importantly, they provide long-term retention of drugs [53], with the ability to encapsulate drugs with limited solubility. The results presented here show that the drug encapsulation efficacy was 86% with a sustained release for more than 40 days at a predetermined rate (Figure 2). This is an important advantage of these systems compared with other nanocarriers such as liposomes that, in general, cannot achieve such high encapsulation efficiency values (recently developed high-pressure processes [54] can achieve drug encapsulation above 90% but are still not common practice). Furthermore, as shown in Figure S2a, these SLPs are retained in the lungs, allowing sustained release of the drug directly in tissues with a high risk of metastasis.

In this study, we encapsulated DOX for different reasons. It is a well-known drug that is commonly used in a variety of cancers; also, its encapsulation contains its undesirable effects such as low specificity to tumor cells, toxic effect in healthy tissues, and an initial burst release followed by a strong decrease in drug concentration when administered on its own [47]. In fact, its clinical use is limited due to its cardiotoxicity and nephrotoxicity [55,56]. We demonstrate that the encapsulation of the drug in SLPs improves the antimetastatic effect of the free drug (Figure 5), improving the biocompatibility in the mice (Figures S5 and S6).

As mentioned above, stable and controlled long-term sustained drug release provides superior therapeutic outcomes than periodically supplied individual higher doses [6,7]. Intravenous administration of conventional drugs usually leads to initial high concentrations followed by a rapid concentration drop below therapeutic limits as a result of drug metabolization or elimination [22]. Thus, our system hypothesizes that the drug release occurs in two well-differentiated steps. The initial one, attributed to the first 5 h of release observed in vitro, occurs while the nanocarrier is circulating in the bloodstream. This allows the elimination of most of the circulating cancer cells. The second, a sustained-release step, takes place upon nanocarrier arrival at the target destination. This surely inhibits tissue colonization by metastatic cells. This drug release pattern would significantly reduce the initial drug dosages and decrease the adverse effects of chemotherapy. Since in the preparation of these solid lipid particles the drug is both integrated within the matrix and adsorbed on the surface of the particles, as concluded by the significant change in the ζ potential from −10 to +23 mV, our system is able to provide this unique two-step drug release effect.

It is well known that mouse models are an important tool to understand the complex mechanism involved in the metastatic process and to identify new targets or improve therapeutic approaches [57]. Previous studies have already established the antitumor efficacy of this kind of formulation in melanoma models [26,27]. Here, we have focused on the effect of SLPs-DOX on metastasis inhibition. To evaluate this antimetastatic effect in vivo, a melanoma lung metastasis model was generated according to Figure 4. This model presents the advantage of producing naked-eye-visible black spots on the lung tissue corresponding to metastatic tumor metastatic colonies.

In previous studies, SLPs have been demonstrated to be effective against metastasis using chemotherapeutic drugs such as paclitaxel [58], etoposide [59], and gallic acid ester derivatives [60], among others. Our results indicate that these particles exhibit a higher antimetastatic effect than unformulated DOX without any detectable toxicity. This antimetastatic effect may be due to the stable drug release over time from these SLPs. This rate of drug release is particularly interesting because it maintains the drug level in blood within the therapeutic window, which is crucial for primary tumor inhibition. This drug release rate in combination with local accumulation in the lungs exerts an improved antimetastatic therapeutic output compared with that of the conventional drug; specifically, SLPs-DOX show 60% more antimetastatic effect than free DOX.

Liposomal doxorubicin formulations (Doxil^®^, Caelyx^®^, and Myocet^®^) are an encapsulated form of doxorubicin, with an improved pharmacokinetic profile and the ability to selectively accumulate in tumor tissue [61]. As a result, the tolerated dose of the drug can be increased, followed by a lower incidence of neutropenia and cardiotoxicity compared with treatment with free doxorubicin. However, the common adverse effect that limits the treatment dose regimen is palmoplantar erythrodysesthesia syndrome [62]. This side effect is a distinctive and relatively common toxic reaction associated with some chemotherapeutic agents. Doxorubicin, cytarabine, docetaxel, and fluorouracil are the agents most frequently implicated. This syndrome appears to be dose dependent, and its occurrence is determined by both the maximum drug concentration and the total cumulative dose. Withdrawal or reduction of the dose of the involved drug usually leads to an improvement in symptoms [63].

In summary, we have demonstrated here the ability to encapsulate DOX in SLPs and evaluated the potential of these particles to release the drug in metastatic tumors in mice. We have shown how the lipid fraction of the particles can significantly improve drug loading and thus generate formulations with improved drug loading and superior stability. We showed how these SLPs allow high retention of the drug in the matrix triggering sustained DOX release upon target tissue arrival [62]. We also demonstrated a significant reduction of the potential adverse effects of the encapsulated DOX, as is the case for the FDA-approved liposomal formulations. However, more interestingly, we demonstrated how the sustained release of the drug upon particle arrival to the target tissue results in an improved antimetastatic effect. This sustained in situ release effect allows improved local efficacy and reduced toxicity compared with that reported for liposomal formulations, where the release is faster and with a higher drug concentration, causing possible adverse effects such as palmoplantar erythrodysesthesia syndrome.

Finally, SLPs can also be prepared with encapsulated iron nanoparticles inside, which in future works will serve not only to trigger on-demand drug release but also for use in imaging techniques or hyperthermia therapy, enhancing the effect of DOX in the tumor [42].

## 5. Conclusions

The goal of this study was to evaluate the metastasis inhibition activity of DOX-loaded SLPs. The results show that these particles synthesized with natural compounds efficiently target lung tissue after only 2 h of intravenous administration. Furthermore, these particles stably release the drug during a long period of over 40 days. This translates into an important reduction in the number of metastases in the lungs compared with unformulated DOX, triggering no detectable toxic effect. The observed antitumor activity in melanoma models [29], together with the metastatic reduction upon treatment with SLPs-DOX, suggest that these particles are an excellent alternative as adjuvant or coadjuvant treatments for numerous systemic cancers.

## Data Availability

Not applicable.

## Supplementary Materials

The following are available online at https://www.mdpi.com/1999-4923/13/1/93/s1, Figure S1. Scheme of the synthesis procedure followed to obtain magnetic DOX-loaded SLPs. Figure S2. Characterization of SLPs. (a) Size distribution of SLP control (left) and SLPs-DOX (right). (b) Transmission electron microscopy image of SLPs. Figure S3. SLPs reach lung tissue. (a) Fluorescent confocal microscopy images in cryostat lungs sections after 3 and 8 h of intravenous administration. The SLPs (green channel) appear close to cell nuclei (stained with Hoechst, blue channel). (b). Inductively coupled plasma spectroscopy (ICP) quantification of Fe in tissues indicative of the biodistribution of the two types of SLPs after 20 days postinjection (*n* = 56). The plot shows the amount of Fe (μg/g) in the liver (L), heart (H), kidneys (K), intestines (I), spleen (S), lungs (Lu), and brain (B). The results were normalized with respect to control tissues (saline serum). Figure S4. Calibration curve of DOX at 597 nm using fluorimetry. Figure S5. Plot of survival rate of mice after 20 days of the experiment; *n* = 167. The results show that SLPs with DOX did not have toxicity at the time of the experiment with respect to untreated mice. Figure S6. Loss of weight mice after 20 days of the experiment; *n* = 13.

## Abbreviations

SLPs	Solid lipid particles
DOX	Doxorubicin
FDA	Food and drug administration
DiO	3,3′-dioctadecyloxacarbocyanine perchlorate
DLS	Dynamic light scattering
TEM	Transmission electron microscopy
HPLC	High-performance liquid chromatography
IMDM	Iscove’s modified Dulbecco’s medium
FBS	Fetal bovine serum
PBS	Phosphate-buffered saline
SEEA	Animal establishment and experimentation service
ICP-OES	Inductively coupled plasma optical emission spectrometry
OCT	Optimum cutting temperature

## References

[1] Gupta G.P., Massagué J. (2006). Cancer Metastasis: Building a Framework. Cell.

[2] Wehner R., Bitterlich A., Meyer N., Kloß A., Schäkel K., Bachmann M., Schmitz M. (2013). Impact of chemotherapeutic agents on the immunostimulatory properties of human 6-sulfo LacNAc+ (slan) dendritic cells. Int. J. Cancer.

[3] Seyfried T.N., Huysentruyt L.C. (2013). On the origin of cancer metastasis. Crit. Rev. Oncog..

[4] Zou L., Wang H., He B., Zeng L., Tan T., Cao H., He X., Zhang Z., Guo S., Li Y. (2016). Current approaches of photothermal therapy in treating cancer metastasis with nanotherapeutics. Theranostics.

[5] Sandru A., Voinea S., Panaitescu E., Blidaru A. (2014). Survival rates of patients with metastatic malignant melanoma. J. Med. Life.

[6] Tas F. (2012). Metastatic behavior in melanoma: Timing, pattern, survival, and influencing factors. J. Oncol..

[7] Weber G.F. (2013). Why does cancer therapy lack effective anti-metastasis drugs?. Cancer Lett..

[8] Morrison V.A. (2014). Immunosuppression associated with novel chemotherapy agents and monoclonal antibodies. Clin. Infect. Dis..

[9] Fidler I.J. (2003). The pathogenesis of cancer metastasis: The “seed and soil” hypothesis revisited. Nat. Rev. Cancer.

[10] Beg S., Rahman M., Jain A., Saini S., Hasnain M.S., Swain S., Imam S., Kazmi I., Akhter S. (2018). Emergence in the functionalized carbon nanotubes as smart nanocarriers for drug delivery applications. Fullerens, Graphenes and Nanotubes.

[11] Parvathy R., Chandran R., Thankam T. (2015). Gold Nanoparticles in Cancer Drug Delivery. Nanotechnology Applications for Tissue Engineering.

[12] Wells A., Grahovac J., Wheeler S., Ma B., Lauffenburger D. (2013). Targeting tumor cell motility as a strategy against invasion and metastasis. Trends Pharmacol. Sci..

[13] Remião M.H., Segatto N.V., Pohlmann A., Guterres S.S., Seixas F.K., Collares T. (2018). The potential of nanotechnology in medically assisted reproduction. Front. Pharmacol..

[14] Tran S., DeGiovanni P.J., Piel B., Rai P. (2017). Cancer nanomedicine: A review of recent success in drug delivery. Clin. Transl. Med..

[15] Wang R., Billone P.S., Mullett W.M. (2012). Nanomedicine in Action: An Overview of Cancer Nanomedicine on the Market and in Clinical Trials. J. Nanomater..

[16] Barenholz Y. (2012). Doxil—The first fda-approved nano-drug: Lessons learned. J. Control. Release.

[17] Lammers T., Hennink W.E., Storm G. (2008). Tumour-targeted nanomedicines: Principles and practice. British J. Cancer.

[18] Chang H.I., Yeh M.K. (2012). Clinical development of liposome-based drugs: Formulation, characterization and therapeutic efficacy. Int. J. Nanomed..

[19] Iturrioz-Rodríguez N., Correa-Duarte M.A., Fanarraga M.L. (2019). Controlled drug delivery systems for cancer based on mesoporous silica particles. Int. J. Nanomed..

[20] Zhou J., Zhang W., Hong C., Pan C. (2015). Silica Nanotubes Decorated by pH-Responsive Diblock Copolymers for Controlled Drug Release. ACS Appl. Mater. Interfaces.

[21] Vallet-Regí M., Colilla M., Izquierdo-Barba I., Manzano M. (2018). Mesoporous Silica Nanoparticles for Drug Delivery: Current Insights. Molecules.

[22] Lee J.H., Yeo Y. (2015). Controlled drug release from pharmaceutical nanocarriers. Chem. Eng. Sci..

[23] Hasan A.S., Socha M., Lamprecht A., El Ghazouani F., Sapin A., Hoffman M., Maincent P., Ubrich N. (2007). Effect of the microencapsulation of nanoparticles on the reduction of burst release. Int. J. Pharm..

[24] Yadav N., Khatak S., Singh Sara U.V. (2013). Solid lipid nanoparticles—A review. Int. J. Appl. Pharm..

[25] Kheradmandnia S., Vasheghani-Farahani E., Nosrati M., Atyabi F. (2010). Preparation and characterization of ketoprofen-loaded solid lipid nanoparticles made from beeswax and carnauba wax. Nanomedicine.

[26] Blum R.H., Carter S.K. (1974). Adriamycin. A new anticancer drug with significant clinical activity. Ann. Intern. Med..

[27] Weiss R.B. (1992). The anthracyclines: Will we ever find a better doxorubicin?. Semin. Oncol..

[28] Carvalho C., Santos R., Cardoso S., Correia S., Oliveira P., Santos M., Moreira P. (2009). Doxorubicin: The Good, the Bad and the Ugly Effect. Curr. Med. Chem..

[29] Jiménez-López J., García-Hevia L., Melguizo C., Prados J., Bañobre-López M.G.J. (2020). Extensive in vitro validation of novel magnetic wax nanocomposite vehicles as cancer combinatorial therapy agents. Evaluation of novel doxorubicin-loaded magnetic wax nanocomposite vehicles as cancer combinatorial therapy agents. Pharmaceutics.

[30] Jadhav N.V., Prasad A.I., Kumar A., Mishra R., Dhara S., Babu K.R., Prajapat C.L., Misra N.L., Ningthoujam R.S., Pandey B.N. (2013). Synthesis of oleic acid functionalized Fe3O4 magnetic nanoparticles and studying their interaction with tumor cells for potential hyperthermia applications. Colloids Surf. B Biointerfaces.

[31] Fidler I.J. (1973). Selection of successive tumour lines for metastasis. Nat. New Biol..

[32] Poste G., Doll J., Hart I.R., Fidler I.J. (1980). In vitro selection of murine B16 melanoma variants with enhanced tissue-invasive properties. Cancer Res..

[33] Overwijk W.W., Restifo N.P. (2000). B16 as a Mouse Model for Human Melanoma. Curr. Protoc. Immunol..

[34] Gautam A., Waldrep J., Densmore C., Koshkina N., Melton S., Roberts L., Gilbert B., Knight V. (2002). Growth inhibition of established B16-F10 lung metastases by sequential aerosol delivery of p53 gene and 9-nitrocamptothecin. Gene Ther..

[35] Bibby M.C. (2004). Orthotopic models of cancer for preclinical drug evaluation: Advantages and disadvantages. Eur. J. Cancer.

[36] Mo R., Jiang T., Gu Z. (2014). Enhanced Anticancer Efficacy by ATP-Mediated Liposomal Drug Delivery. Angew. Int. Ed. Engl..

[37] Dicheva B.M., ten Hagen T.L.M., Seynhaeve A.L.B., Amin M., Eggermont A.M.M., Koning G.A. (2015). Enhanced Specificity and Drug Delivery in Tumors by cRGD—Anchoring Thermosensitive Liposomes. Pharm. Res..

[38] Honig M.G., Hume R.I. (1989). Dil and DiO: Versatile fluorescent dyes for neuronal labelling and pathway tracing. Trends Neurosci..

[39] Von Bartheld C.S., Cunningham D.E., Rubel E.W. (1990). Neuronal tracing with DiI: Decalcification, cryosectioning, and photoconversion for light and electron microscopic analysis. J. Histochem. Cytochem..

[40] Weber S., Zimmer A., Pardeike J. (2014). Solid Lipid Nanoparticles (SLN) and Nanostructured Lipid Carriers (NLC) for pulmonary application: A review of the state of the art. Eur. J. Pharm. Biopharm..

[41] Swainson S.M.E., Taresco V., Pearce A.K., Clapp L.H., Ager B., McAllister M., Bosquillon C., Garnett M.C. (2019). Exploring the enzymatic degradation of poly(glycerol adipate). Eur. J. Pharm. Biopharm..

[42] Iglesias G.R., Reyes-Ortega F., Checa Fernández B.L., Delgado A.V. (2018). Hyperthermia-triggered gemcitabine release from polymer-coated magnetite nanoparticles. Polymers.

[43] Hu X., Tian J., Liu T., Zhang G., Liu S. (2013). Photo-triggered release of caged camptothecin prodrugs from dually responsive shell cross-linked micelles. Macromolecules.

[44] Kundu J.K., Surh Y.J. (2010). Nrf2-Keap1 signaling as a potential target for chemoprevention of inflammation-associated carcinogenesis. Pharm. Res..

[45] Kaddha S., Khreich N., Kaddah F., Charcosset C., Greige-Gerges H. (2018). Chloresterol modulates the liposome membrane fluidity and permeability for a hydrophilic molecule. Food Chem. Toxicol..

[46] Korsmeyer R.W., Gurny R., Doelker E., Buri P., Peppas N.A. (1983). Mechanisms of solute release from porous hydrophilic polymers. Int. J. Pharm..

[47] Kashanian S., Azandaryani A.H., Derakhshandeh K. (2011). New surface-modified solid lipid nanoparticles using N-glutaryl phosphatidylethanolamine as the outer shell. Int. J. Nanomed..

[48] De Almeida R.R., Gallo J., Da Silva A.C.C., Da Silva A.K.O., Pessoa O.D.L., Araújo T.G., Leal L.K.A.M., Fechine P.B.A., Bañobre-López M., Ricardo N.M.P.S. (2017). Preliminary Evaluation of Novel Triglyceride-Based Nanocomposites for Biomedical Applications. J. Braz. Chem. Soc..

[49] Wen H., Jung H., Li X. (2015). Drug Delivery Approaches in Addressing Clinical Pharmacology-Related Issues: Opportunities and Challenges. AAPS J..

[50] Weisser J.R., Saltzman W.M. (2014). Controlled release for local delivery of drugs: Barriers and models. J. Control. Release.

[51] Mitragotri S., Burke P.A., Larger R. (2014). Overcoming the challengues in administering biopharmaceuticals: Formulation and delivery strategies. Nat. Rev. Drug Discov..

[52] Mellema M., Van Benthum W.A.J., Boer B., Von Harras J., Visser A. (2006). Wax encapsulation of water-soluble compounds for application in foods. J. Microencapsul..

[53] De M. Barbosa R., Ribeiro L.N.M., Casadei B.R., Da Silva C.M.G., Queiróz V.A., Duran N., de Araújo D.R., Severino P., De Paula E. (2018). Solid Lipid Nanoparticles for Dibucaine Sustained Release. Pharmaceutics.

[54] Trucillo P., Campardelli R., Reverchon E. (2020). Liposomes: From bangham to supercritical fluids. Processes.

[55] Bae Y., Diezi T.A., Zhao A., Kwon G.S. (2007). Mixed polymeric micelles for combination cancer chemotherapy through the concurrent delivery of multiple chemotherapeutic agents. J. Control. Release.

[56] Kakinoki A., Kaneo Y., Ikeda Y., Tanaka T., Fujita K. (2008). Synthesis of poly(vinyl alcohol)-doxorubicin conjugates containing cis-aconityl acid-cleavable bond and its isomer dependent doxorubicin release. Biol. Pharm. Bull..

[57] Gomez-Cuadrado L., Tracey N., Ma R., Qian B., Brunton V.G. (2017). Mouse models of metastasis: Progress and prospects. DMM Dis. Model. Mech..

[58] Ma P., Benhabbour S.R., Feng L., Mumper R.J. (2013). 2′-Behenoyl-paclitaxel conjugate containing lipid nanoparticles for the treatment of metastatic breast cancer. Cancer Lett..

[59] Athawale R.B., Jain D.S., Singh K.K., Gude R.P. (2014). Etoposide loaded solid lipid nanoparticles for curtailing B16F10 melanoma colonization in lung. Biomed. Pharmacother..

[60] Cordova C.A.S., Locatelli C., Winter E., Silva A.H., Zanetti-Ramos B.G., Jasper R., Mascarello A., Yunes R.A., Nunes R.J., Creczynski-Pasa T.B. (2017). Solid lipid nanoparticles improve octyl gallate antimetastatic activity and ameliorate its renal and hepatic toxic effects. Anticancer Drugs.

[61] Waterhouse D.N., Tardi P.G., Mayer L.D., Bally M.B. (2001). A comparison of liposomal formulations of doxorubicin with drug administered in free form: Changing toxicity profiles. Drug Saf..

[62] Stella B., Peira E., Dianzani C., Gallarate M., Battaglia L., Gigliotti C.L., Boggio E., Dianzani U., Dosio F. (2018). Development and Characterization of Solid Lipid Nanoparticles Loaded with a Highly Active Doxorubicin Derivative. Nanomaterials.

[63] Lorusso D., Di Stefano A., Carone V., Fagotti F., Pisconti F., Scambia G. (2007). Pegylated liposomal doxorubicin-related palmar-plantar erythrodysesthesia (‘hand-foot’ syndrome). Ann. Oncol..

