# Supplementary material for: Solid Lipid Particles for Lung Metastasis Treatment
Source: Pharmaceutics. 2021 Jan 13;13(1):93. doi: 10.3390/pharmaceutics13010093 (PMC7828486; doi:10.3390/pharmaceutics13010093)
Supplement: Supplementary file 1 [file pharmaceutics-13-00093-s001.pdf]

pharmaceutics

MDPI

Supplementary Materials: Solid lipid particles for lung metastasis treatment

Lourdes Valdivia, Lorena García-Hevia, Manuel Bañobre-López, Juan Gallo, Rafael Valiente and Mónica López Fanarraga

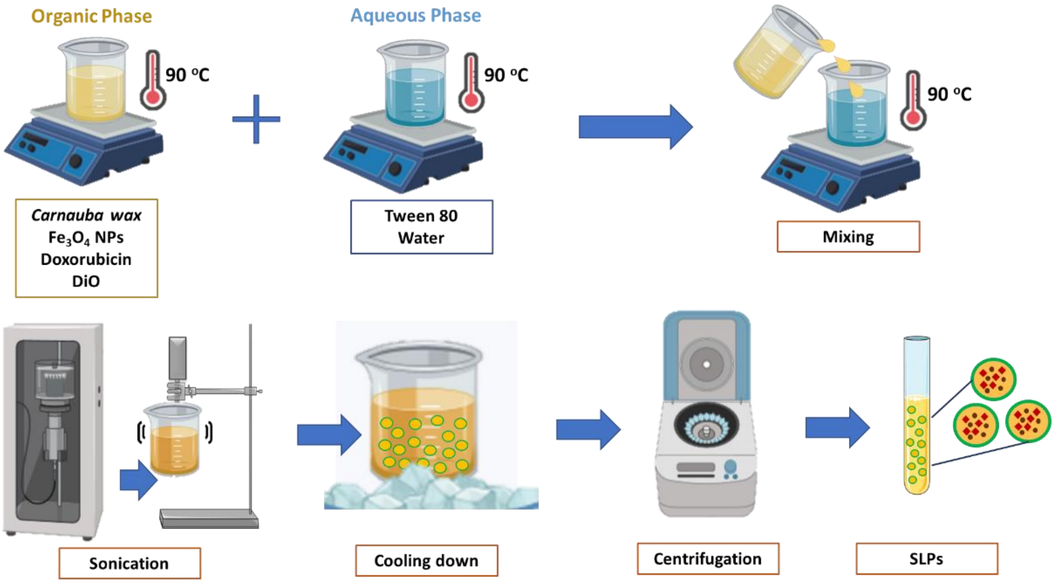

Figure S1. Scheme of the synthesis procedure followed to obtain magnetic DOX-loaded SLPs.

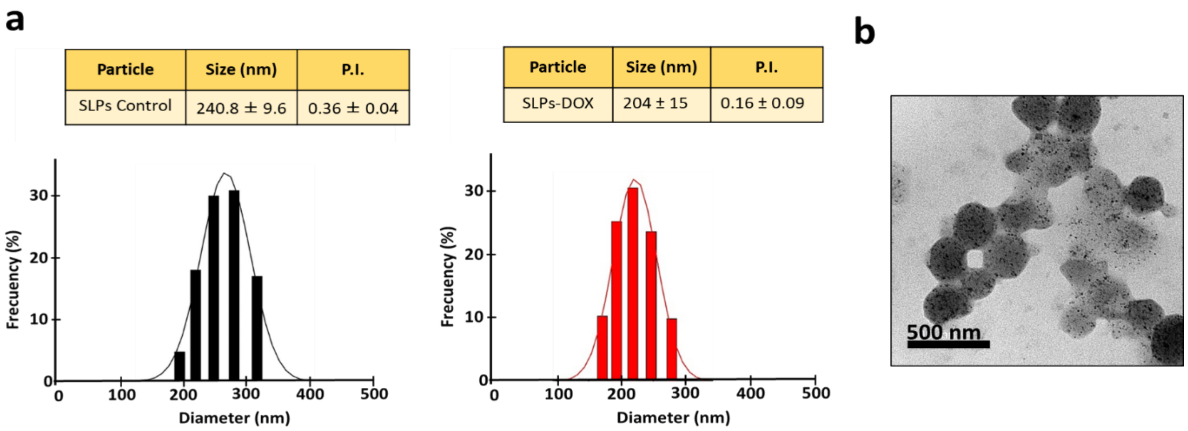

Figure S2. Characterization of SLPs. **(a)** Size distribution of SLPs control (left) and SLPs-DOX (right). **(b)** Transmission electron microscopy image of SLPs.

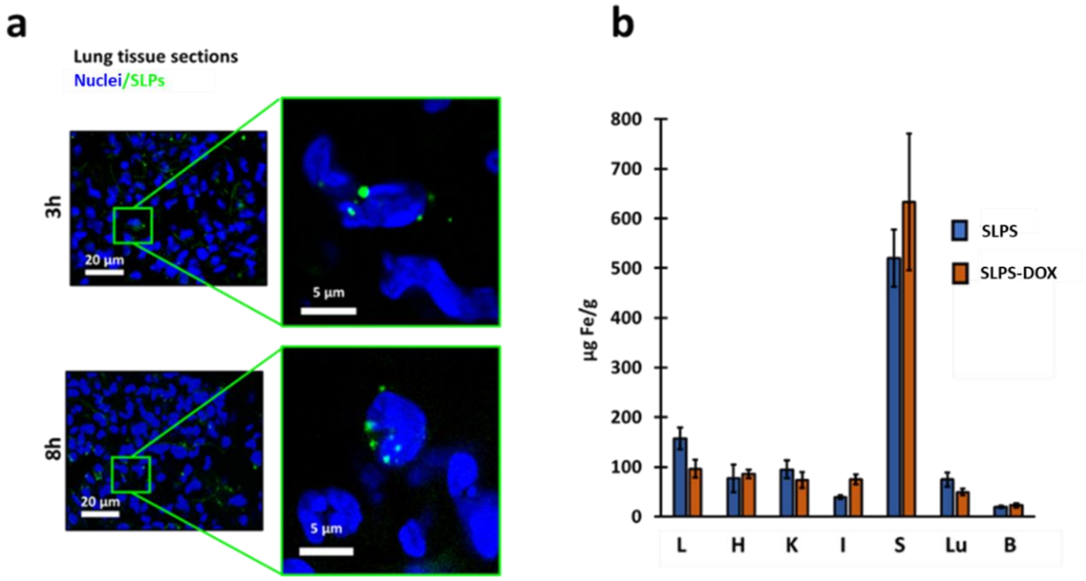

Figure S3. SLPs reach lung tissue. **(a)** Fluorescent confocal microscopy images in cryostat lungs sections after 3h and 8h of intravenous administration. The SLPs (green channel) appear close to cell nuclei (stained with Hoechst, blue channel). **(b)**. Inductively coupled plasma spectroscopy (ICP) quantification of Fe in tissues indicative of the biodistribution of the two types of SLPs after 20 days post-inyection (n = 56). The plot shows the amount of Fe (µg/g) in the liver (L), heart (H), kidney (K), intestine (I), spleen (S), lungs (Lu) and brain (B). The results were normalized respect to control tissues (saline serum).

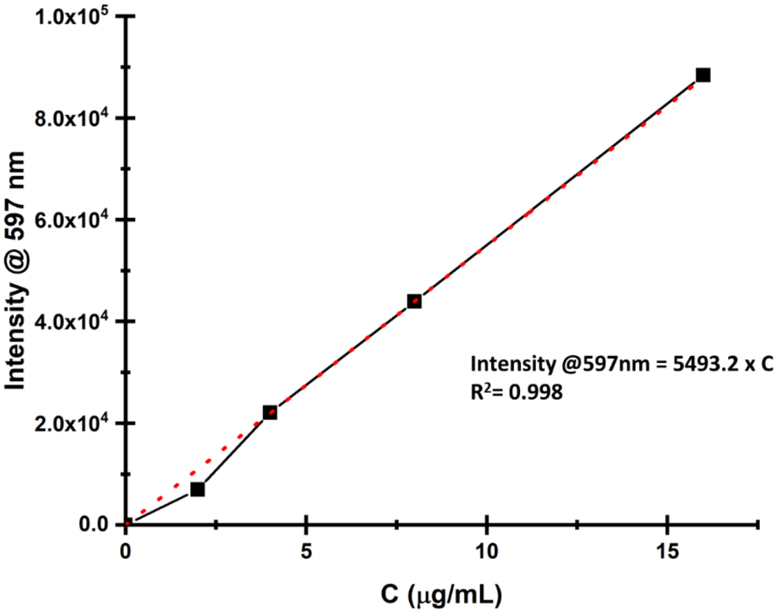

Figure S4. Calibration curve of DOX at 597 nm using fluorimetry.

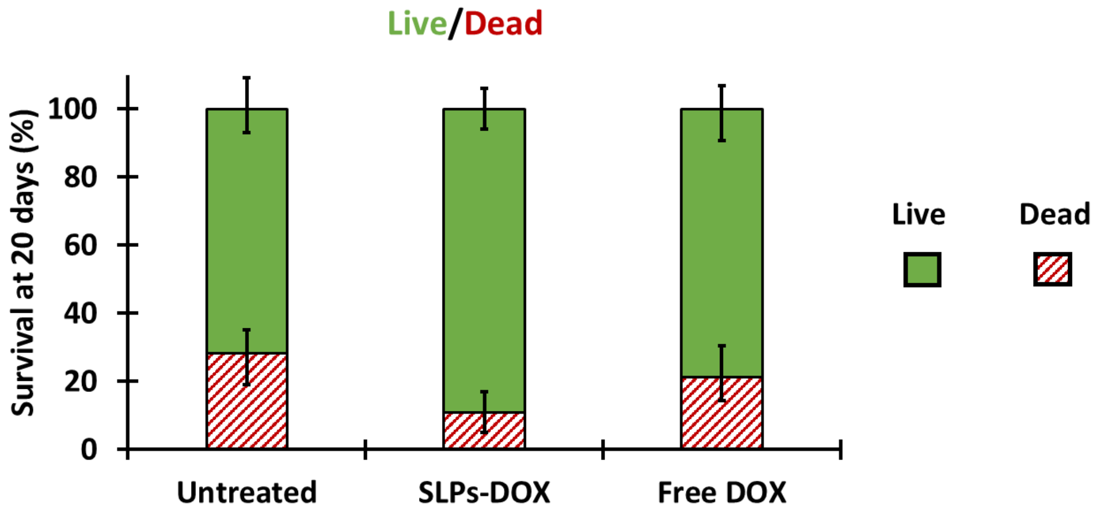

Figure S5. Plot of survival rate of mice after 20 days of the experiment; n = 167. The results show that SLPs with DOX not have toxicity in the time of the experiment respect to untreated mice.

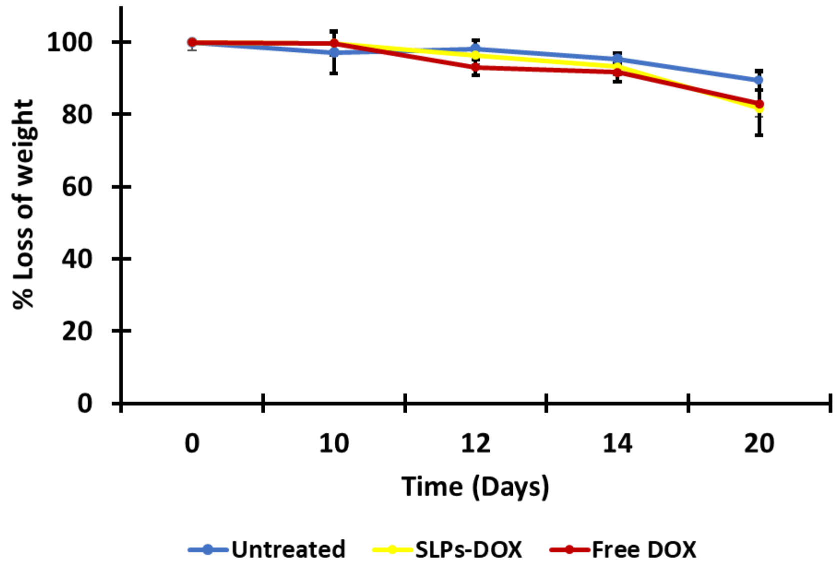

Figure S6. Loss of weight mice after 20 days of the experiment; n = 13.
